# Identification and Antimicrobial Potential of Marine Sponges (*Carteriospongia foliascens*, *Callyspongia fallax*, and *Paratetilla arcifera*) from Kenyan Marine Waters

**DOI:** 10.1155/ijm/4208163

**Published:** 2025-09-03

**Authors:** Teresia Nyambura Wacira, Huxley Mae Makonde, Joseph Nyingi Kamau, Cromwell Mwiti Kibiti

**Affiliations:** ^1^Department of Pure and Applied Sciences, Technical University of Mombasa, Mombasa, Kenya; ^2^Kenya Marine and Fisheries Research Institute, Freshwater Research Center, Kisumu, Kenya; ^3^Kenya Marine and Fisheries Research Institute, Mombasa, Kenya

**Keywords:** marine bioactive compounds, marine natural products, metazoan sponges, sponge biochemicals

## Abstract

Emerging and re-emerging infectious diseases and pathogens present a significant global public health threat that has led researchers to focus on discovering new antimicrobial agents in order to address the challenge. Sponges are a promising source of marine natural products, which can be used as lead molecules for drug discovery. This study was aimed at identifying marine sponges through morphological and molecular techniques and evaluate the bioactivity potential of their organic crude extracts against *Escherichia coli*, *Staphylococcus aureus*, *Pseudomonas aeruginosa*, and *Candida albicans.* Deoxyribonucleic acid (DNA) barcoding of the cytochrome c oxidase subunit I (COI) gene identified three genera of sponges (*Carteriospongia*, *Callyspongia*, and *Paratetilla*). Disk diffusion assay was used to determine the inhibition zone diameter (IZD) of the sponges' extracts. Minimum inhibitory concentrations (MICs) and the minimum bactericidal/fungicidal concentrations (MBCs/MFCs) of the most active sponge extracts were determined. The bioactive compounds were analyzed using gas chromatography–mass spectrometry (GC-MS). The dichloromethane extracts of *Carteriospongia foliascens* demonstrated the highest antifungal activity against *C. albicans* (31.33 ± 1.2 mg mL^−1^), surpassing the standard drug fluconazole (29.33 ± 1.5 mg mL^−1^). The MIC values for the sponge extracts ranged from 3.86 to 5.89 mg mL^−1^, and the ethyl acetate extract of *Callyspongia fallax* had an MBC of 4.03 mg mL^−1^ against *S. aureus*. GC-MS chromatogram identified 98 compounds across 41 classes in three sponge extracts. Notably, 9.2% of these compounds have been reported to exhibit antimicrobial activity against human pathogens. This study confirms that sponges are a source of useful biochemicals, which have potential for drug discovery. To the best of our knowledge, this is the first comprehensive study to report on the characterization of marine sponges from the Kenyan waters. Further research work to structurally elucidate and identify the most active bioactive compounds from the extracts of *C. foliascens* and *C. fallax* is recommended.

## 1. Introduction

The vast biodiversity of marine ecosystem communities, including algae, bacteria, sea cucumbers, fungi, and sponges, continues to be tapped to discover novel compounds that can overcome microbial resistance, which is a growing concern in the pharmaceutical industry [1]. Discovery of new enzymes, natural products, and genes in order to enhance the commercial production of drugs is important because of the development of drug resistance by pathogenic microbes [2].

Marine sponges are pivotal in the cycling of nutrients and organic matter in oceanic ecosystems and are reported to be a significant source of bioactive substances [3]. Notably, the class Demospongiae is an opulent source of biologically active specialized biochemical compounds with potential applications, including antimicrobial, antitumor, antiviral, and antifouling properties [4]. Moreover, sponge preferences and features are crucial in analyzing their environment [5] and reconstructing oscillations in pH, water depths, temperatures, and other parameters, providing historical climate snapshots [6]. Spicules provide sponge skeletal stability for holding the body erect, reduce the metabolic expenditure required for water circulation, and can additionally repel predators [6].

Demosponges are unique in their ability to methylate sterols at the 26-position, which aids in identifying their presence even before the appearance of unambiguous fossils [7]. Marine sponges also produce allelochemicals, biochemical agents used to deter predators, prevent overgrowth by fouling organisms, and protect against pathogens [8]. Consequently, marine sponges are a treasure trove of bioactive compounds with potential applications in medicine [9]. Metazoan sponges have been reported to yield a variety of chemical groups with promising antimicrobial activities, including alkaloids, diacylglycerols, polysaccharides, terpenoids, steroids, polyketides, and peptides [10]. Research indicates that the order Dictyoceratida of marine sponges produces a range of terpenes and alkaloids with notable antimicrobial properties [11]. Verongida sponges produce bromotyrosine derivatives with significant antifungal and antibacterial activities [12]. A similar research study investigated the antimicrobial properties of the sponge *Axinella infundibuliformis*, collected from Mombasa, Kenya. The sponge yielded 3*β*-hydroxylup-20 (29)-ene, which demonstrated the highest antibacterial activity against *Pseudomonas aeruginosa*, surpassing the standard drug gentamycin [13].

Exploration of novel bioactive compounds from marine sponges could lead to breakthroughs in medical science, particularly in the development of new treatments for infections that are currently difficult to treat [14]. Along the African coasts, sponges have not been adequately identified and exploited despite their useful value as indicators of environmental health. In order to extensively explore the Kenyan marine sponges, the current study used both morphological and molecular techniques for taxonomic identification, a disk diffusion method for bioassay analysis, and gas chromatography–mass spectrometry (GC-MS) to identify and quantify different compounds in the extracts of the marine sponge samples. The study findings provide additional insights into the bioactive compounds repertoire of sponges that have potential application in drug discovery.

## 2. Materials and Methods

### 2.1. Ethical Statement

This research study received official authorization from the National Commission for Science, Technology and Innovation (NACOSTI), Kenya, under License No.: NACOSTI/P/25/4174598. Ethical clearance was obtained from the Technical University of Mombasa (TUM), Approval No.: TUM SERC PhD/006/2025, affirming adherence to institutional standards for ethical research conduct. Access to field sampling sites was granted by the Kenya Marine and Fisheries Research Institute (KMFRI) through Reference No.: GOK_PC Target C82 39-1/22, ensuring compliance with environmental research regulations. Importantly, the fieldwork did not involve endangered or protected species, in accordance with national conservation protocols and ethical research principles.

### 2.2. Study Sites

**Figure 1:**
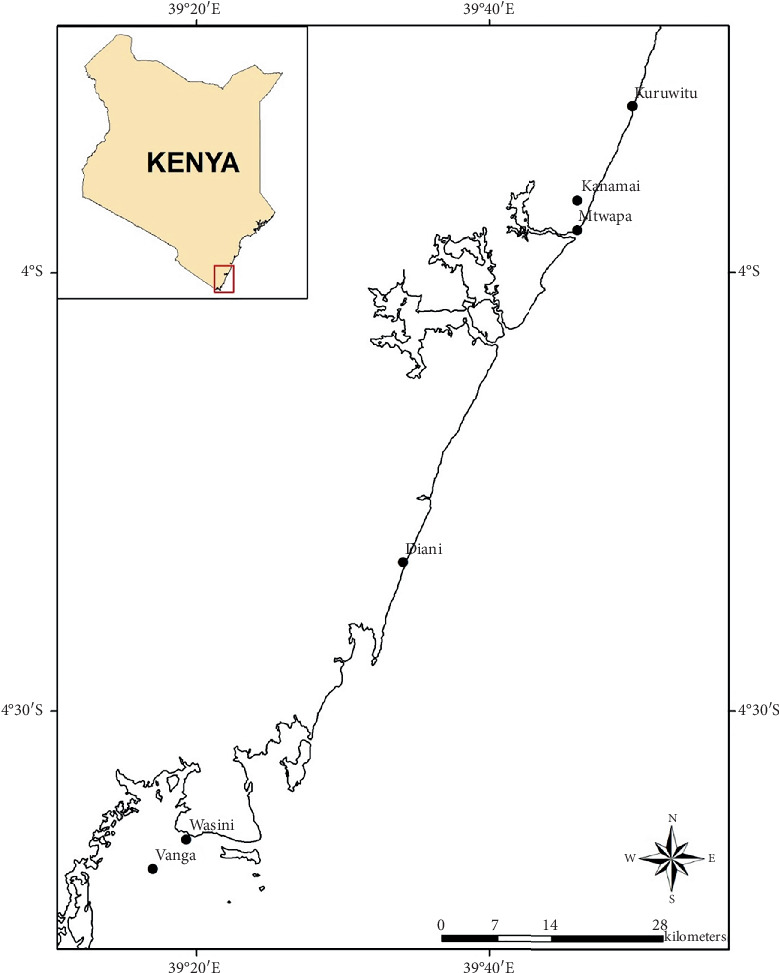
Map of the coastline of Kenya showing the locations of selected study sites: northern coastline (Kuruwitu, Kanamai, and Mtwapa) where the marine sponges were collected.

The sponges were collected from three hotspot sites (at the Kenyan Northern coastline, including Mtwapa (3°57⁣′07.1⁣^″^ S 39°46⁣′05.9⁣^″^ E), Kuruwitu (3°48⁣′39.6⁣^″^ S 39°49⁣′52.0⁣^″^ E), and Kanamai (3°55⁣′ S 39°46⁣′ E), which provide a diverse range of habitats from seagrass meadows to mangrove forests (Figure 1).

### 2.3. Sampling and Sample Processing

An initial survey was conducted at each site to assess the abundance and availability of sponges, during which the species present and their population densities were recorded. Based on the survey results, sponges were purposively sampled to ensure a diverse representation of the sponge species [15, 16]. A total of 23 marine sponge samples were collected from six study sites along the northern and southern coastlines of Kenya, encompassing a diverse range of taxa with varying abundance and distribution patterns (Tables S1–S3). For this specific study, three sponge species were selected for detailed analysis, each representing a distinct abundance category and collected from separate sites along the northern coastline (Kuruwitu, Kanamai, and Mtwapa). These included *Carteriospongia foliascens* (high abundance, observed at more than four sites), *Callyspongia fallax* (moderate abundance, recorded at two to three sites), and *Paratetilla arcifera* (rare, found at a single site). The sponges were collected during the southeast monsoon within a 60 min SCUBA dive, navigating through the rocky reefs at a depth range of 4.8–18 m [17].

The marine sponges were coded depending on the lifeform color and the study site collected; the prefixes CY, BR, and GY represented cream yellow, brown, and gray, respectively. The prefix was followed by the letters (Ku, M, and Ka) representing Kuruwitu, Mtwapa, and Kanamai, respectively, and the specimen number of the sponge collected from the coastline of Kenya. Protective gloves were used to ensure the safety of the researcher, as well as to prevent contamination of the sponge samples [18]. Using a knife, the sponges were gently cut from their substrate to preserve their structure and integrity [19]. Once collected, each sponge's characters were documented with in situ photographs that captured their unique characteristics. Detailed notes on surface features, locality, color, and habitat were also recorded, following the protocols outlined by [20, 21] before the samples were analyzed for their biodiversity and potential bioactive compounds.

The marine sponges were rinsed twice with sterile seawater, and large sections of about 10 cm^3^ each from each sample were packed into labeled sterile zip lock bags, placed in a cool box with ice packs, and frozen at −80°C for antimicrobial assays screening [22]. The other section was preserved in 96% ethanol for sponge morphological identification [23]. Following collection, the samples were preserved in a cooler with dry ice and transported to the TUM and KMFRI laboratories in Mombasa, Kenya, where they were immediately refrigerated for further analysis.

### 2.4. Marine Sponge Morphological Identification

#### 2.4.1. Spicule Preparations

Small pieces of sponges (about 4 cm^3^) were immersed in a bleach solution. The spicules were allowed to settle for 10–15 min after the skeleton's sponging tissue dissolved in sodium hypochlorite, allowing organic components to dissolve and leaving only the mineral skeleton [20]. The spicule solution was rinsed thrice with distilled water to eliminate the bleach before being preserved in 70% ethanol [24]. The clean spicule suspensions were aspirated and pipetted onto a microscope glass slide, followed by the evaporation of the ethanol [6]. The remaining sample was observed under a Primo Star ZEISS, Jena, Germany, image analyzer microscope while covered with a coverslip. Microphotographs were taken with an Axio cam camera ERc5s digital camera (Carl Zeiss, Germany).

#### 2.4.2. Skeleton Preparations

The sponge specimens were sliced using a sterile scalpel into approximately 30–200 *μ*m for skeleton observations and mounted on microscopic slides for observation under a dissecting microscope (Amscope LED-144S, China) [25]. The Sponge Identification Reference Book by [26] was utilized alongside the Porifera database list (http://www.marinespecies.org/porifera/) to determine the identity of the genus and species of the marine sponges [27]. The taxonomy of the marine species was done using the World Register of Marine Species (WoRMS) (https://www.marinespecies.org/) database [28].

#### 2.4.3. Deoxyribonucleic Acid (DNA) Extraction From Marine Sponge Using Chelex Resin

The specimens were rinsed with sterile ocean water to remove any surface contaminants. The sponges were cut into smaller pieces and placed in a microcentrifuge tube along with a Chelex resin suspension [29]. The mixture was heated for 30 min to facilitate the lysis of cells, allowing the DNA to be released into the solution. The microcentrifuge tubes were spun at high speed, which allowed the Chelex resin to pellet at the bottom while the DNA remained in the supernatant [30]. The supernatant, containing the DNA, was transferred to a new tube, and the DNA concentration and purity were assessed using a NanoDrop spectrophotometer for downstream applications. The targeted amplification of the marine sponges was achieved using the gene-coding mitochondrial cytochrome oxidase subunit I (COI) with a length of 640 bp [31] using the following pair of primers: dgLCO1490: GGT CAA CAA ATC ATA AAG AYA TYG G and dgHCO2198: TAA ACT TCA GGG TGA CCA AAR AAY CA [32].

A master mix comprising 0.2 mM dNTPs, 1X PCR buffer, 1.5 mM MgCl_2_, and 0.05 *μ*M Taq DNA polymerase was prepared. Each sample's PCR reaction mixture consisted of 4 *μ*L of Milli-Q water, 10 *μ*L of 2X master mix, and 2 *μ*L each of the 0.2 *μ*M COI primers (forward and reverse). After excluding the Taq polymerase, the mix was centrifuged, and 18 *μ*L was dispensed into each PCR tube. Subsequently, 2 *μ*L of genomic DNA template was added to each tube. The individual PCR components were combined to reach a final volume of 20 *μ*L in each tube. The integrity of the PCR amplicons was assessed using a 1% agarose gel (CSL-AG500, Cleaver Scientific Ltd) stained with EZ-Vision Bluelight DNA Dye (Lorenz, 2012). The FastDNA Ladder-NEB served as the DNA marker, and distilled water was used as both the positive and negative control [33].

For sequencing, the PCR amplicons were sent to a commercial service provider, Inqaba Biotec Company in South Africa. The sequencing procedure involved the utilization of the Nimagen BrilliantDye Terminator Cycle Sequencing Kit V3.1, BRD3-100/1000, following the manufacturer's stipulated guidelines [34]. The labeled products were then purified using the ZR-96 DNA Sequencing Clean-up Kit before being injected on an Applied Biosystems ABI 3730XL Genetic Analyzer with a 50 cm array, using POP7 [35]. Subsequently, the sequence chromatogram analysis was conducted using FinchTV analysis software to visualize and interpret the DNA sequence data.

The sequencing results were analyzed by employing established bioinformatics tools. Initially, the BioEdit Sequence Alignment Editor (Version 7.7) was utilized [36] to edit and trim to obtain complete DNA sequences. Subsequently, the sequences were subjected to analysis using the Basic Local Alignment Search Tool (BLAST) to search for similar sequences at the National Center for Biotechnology Information (NCBI) platform GenBank (https://www.ncbi.nlm.nih.gov/nucleotide/) [37]. The closest nucleotide sequences were retrieved from the GenBank database and saved in FASTA format, along with the newly obtained sequences from the study. The Clustal Omega program (http://www.clustal.org) was used to align all the sequences against their nearest neighbors [38].

The MEGA X (Molecular Evolutionary Genetics Analysis) software platform was utilized to construct a neighbor-joining tree of the aligned sequences [39]. Evolutionary distances were computed utilizing the maximum composite likelihood method. To evaluate the statistical robustness of the phylogenetic branches, a bootstrap analysis with 1000 replicates was conducted [40]. In the course of the phylogenetic analysis, all sequence alignment sites, including gaps, were pairwise excluded. Each isolate was subsequently categorized into its corresponding taxonomic group based on the resultant neighbor-joining tree [39]. Further validation of the taxonomic classification was achieved at a 90% confidence level through the application of the naïve Bayesian rRNA classifier available on the Ribosomal Database Project (RDP) website [41].

#### 2.4.4. Preparation of the Sponge Extracts

Each marine sponge sample (500 g wet weight) was cut into small pieces, mixed with a blender, and macerated at 4°C for 48 h [42]. The macerate was lyophilized for 3 days using a laboratory freeze dryer (ULVAC Technologies, Methuen, Japan) with methanol as the solvent. After lyophilization, 100 g of the powdered sponge material was sequentially extracted with methanol, dichloromethane (DCM), and ethyl acetate (EtOAc), following standard cold maceration procedures. Each extraction step involved agitation of the material in 500 mL of the respective solvent at room temperature for 48 h. The solvent fractions were concentrated using a rotary evaporator at 40°C under reduced pressure. The yields obtained (*w*/*w*) were as follows: methanol extract 6.2%, DCM extract 4.8%, and EtOAc extract 3.5%.

The samples were then dried under a rotary vacuum evaporator (BIOBASE Company, Jiangsu, China) and screened against three human pathogenic strains: *Staphylococcus aureus* ATCC 25923, *P. aeruginosa* ATCC 27853, *Escherichia coli* ATCC 25922, and one human pathogenic fungus, *Candida albicans* ATCC 10231 strain, using the agar disk diffusion method [43]. All the bacteria and *C. albicans* strains were obtained from the Microbiology Department of the Kenya Medical Research Institute (KEMRI), Kenya.

### 2.5. Antibacterial and Antifungal Screening of Marine Sponge Crude Extracts

The disk diffusion method was used to screen for antibacterial properties [44]. The assay utilized the Mueller–Hinton agar (MHA) (TM Media, India), which was prepared according to the manufacturer's instructions to determine antibacterial activity [45]. Mueller–Hinton broth (MHB) (HiMedia, Mumbai, India) was prepared according to the manufacturer's instructions. The bacterial test organisms were incubated overnight in MHB at 37°C with agitation at 150 rpm [46].

The evaluation of bacterial proliferation commenced with the measurement of optical density (OD) at 600 nm [47]. A culture of the test organisms was standardized to an OD of 0.25 using a biological density meter (Biomerieux VITEK Densichek plus Analyzer, United States). The standardized culture was then streaked onto the MHA plates using a sterile swab and a Rota-plater (Etest Inoculator Retro C80, bioMerieux, United States). The MHA plates with the test organisms were left to air dry [48]. To verify the efficacy of marine sponge extracts using the disk assay method, triplicate tests were conducted with methanolic, DCM, and EtOAc extracts. To assess their activity, these sponge extracts (10 *μ*L of 10 mg mL^−1^ sponge extracts in dimethyl sulfoxide [DMSO]) were infused [49] into sterile 6 mm Whatman Antibiotic Assay Discs (Sigma-Aldrich Company, Germany) [50]. DMSO served as the negative control, while vancomycin (200 *μ*g/mL) and ciprofloxacin (200 *μ*g/mL) were employed as positive controls to validate experimental results against Gram-positive and Gram-negative bacteria, respectively [51]. After the establishment of the controls and marine sponge extracts, the bacterial culture was incubated overnight at 37°C [52]. Subsequently, the resulting zone of inhibition surrounding the disks was measured in millimeters using an electronic vernier caliper according to the Clinical and Laboratory Standards Institute (CLSI-2020) guidelines [53].

In the assessment of antifungal activity, potato dextrose broth (PDB) (TM Media, India) was used for the seed medium, and potato dextrose agar (PDA) (TM Media, India) was used for antifungal assays. The preparations involved suspending 24 g of PDB and 39 g of PDA in 1 L of distilled water, respectively [54]. Fluconazole (10 *μ*g/mL) served as the positive control [55]. DMSO was used to dissolve the sponge extracts and served as the negative control. The experiments were conducted in three independent replicates to ensure the reliability and reproducibility of the results.

### 2.6. Minimum Inhibitory Concentrations (MICs) and the Minimum Bactericidal/Fungicidal Concentrations (MBCs/MFCs) of the Sponge Extracts

The lowest concentration of the drugs that inhibited the growth of the test microorganisms was determined using the broth microdilution assay [56]. The bacterial cultures used as the test microorganisms in the antibacterial screening were prepared using MHB. The OD of the bacterial culture was adjusted to 0.5 = 1 × 10^8^ CFU/mL using the McFarland Standard [57]. The antifungal, antibiotic drugs, and sponge-derived antimicrobial extracts were diluted using a series of concentrations. This process involved creating a gradient of the stock solution in nutrient broth, with decreasing concentrations: 10, 5, 2.5, 1.25, and 0.625 mg mL^−1^ [58]. The different concentrations were transferred into a 96-well plate for inoculation, and two controls were used [59]. The growth control that contained no drug or antimicrobial extract, but just the test microorganism, was expected to show how the bacteria grow without the extract [60]. The sterile control had only the nutrient broth and did not include any test microorganisms. Its purpose was to ensure the reliability of the results by confirming the sterility of the media, culturing plates, and equipment used in the experiment [61]. The incubation was done over approximately 18 h at 37°C. The UV-VIS spectrophotometer (Konik, Barcelona, Spain) was used to measure the turbidity at a wavelength of 620 nm [62].

The MBC/MFC was recorded as the lowest concentration at which no bacterial or fungal growth was observed on the agar plates after the determination of the MIC dilution [63]. To determine the MBC/MFC, the MIC dilution and at least two dilutions with higher concentrations of the standard drug and the sponge organic crude extracts (100 *μ*L) were cultured into the fresh agar plates [64]. The agar plates were incubated at 37°C for 18 h for bacterial cultures, while *C. albicans* cultures were incubated at 30°C for 48 h. The MBC/MFC of the lowest concentration on the agar plates where bacterial or fungal growth was completely inhibited was recorded [65].

### 2.7. GC-MS Analysis of the Marine Sponge Crude Extracts

The GC-MS analysis was performed using a GCMS-TQ8040 NX quadrupole (Shimadzu, Eurasia), fused with SH-I-5MS silica capillary column (30 m × 0.25 mm × 0.25 *μ*m film thickness) [66]. Helium was used as the carrier gas at a constant flow rate of 1 mL/min, with a sample injection volume of 1 *μ*L. The temperature program began with the injector at 250°C and the oven at 110°C, rising to 200°C at 10°C/min, then to 280°C at 5°C/min, holding at 280°C for 9 min [67]. The sponge extracts were filtered through 0.22 *μ*m polytetrafluoroethylene (PTFE) membranes (Merck Millipore, Germany) to remove particulates, then analyzed by GC-MS using split-mode injection to prevent column overloading and improve sample delivery into the gas chromatograph, thereby enhancing the sensitivity for detecting bioactive compounds [68].

The initial oven temperature was set at 50°C to allow for the efficient volatilization of low-boiling-point components [69]. A controlled temperature ramp was then applied, gradually increasing the oven temperature to 300°C at a predefined rate. This temperature gradient facilitated the effective separation of compounds with varying volatilities, ensuring optimal resolution and peak identification during GC-MS analysis [70]. The final temperature of 300°C was maintained for a specific duration to allow the elution of high-molecular-weight compounds, enhancing the accuracy and reliability of compound identification [71]. The separated compounds were introduced into the mass spectrometer, where ions were analyzed by their mass-to-charge ratio (*m*/*z*), generating unique mass spectra. These spectra were compared with the National Institute of Standards and Technology (NIST) 14 library for compound identification [72].

### 2.8. Data Analysis

The statistical data analysis was performed using a two-way ANOVA using Minitab Statistical Software Version 22.1.0. The data was presented as mean ± standard deviation (SD) for the inhibition zones. To ensure that only significant differences are considered, Fisher's least significant difference (LSD) test was applied at a 95% confidence interval. To minimize the probability of Type I errors, a *p* value of less than 0.05 was set as the threshold for significance.

The mass spectra of each compound obtained from GC-MS analysis were compared against entries in the NIST 14 Mass Spectral Library; only those with high match quality scores (above 75%) were considered for interpretation. Where applicable, these tentative identifications were further validated by cross-referencing published literature and retention index data.

## 3. Results

### 3.1. Marine Sponge Morphology and Taxonomy

During the initial survey of marine sponge sampling sites, Mtwapa showed higher diversity and abundance of sponges compared to Kuruwitu and Kanamai. Three metazoan marine sponge species were collected in this study. The voucher specimens CYKu 002 (Kuruwitu), GYM 009 (Mtwapa), and BRKa 005 (Kanamai) were deposited at the KMFRI museum department. The metazoan sponges were taxonomically identified as *C. foliascens*, *C. fallax*, and *P. arcifera*, respectively.

**Figure 2:**
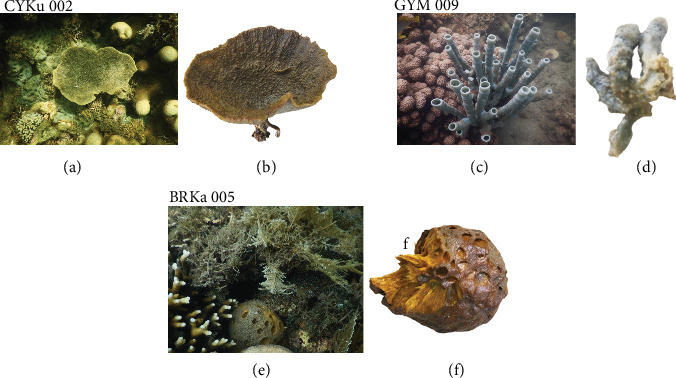
In situ ocean underwater and substrate-detached marine sponge photographs collected from Kenya's northern coast (*Source:* Author). Key: (a) *Carteriospongia foliascens* (voucher specimen [CYKu 002] in underwater), (b) *C. foliascens* after substrate-detachment, (c) *Callyspongia fallax* (voucher specimen [GYM 009] in underwater), (d) *C. fallax* after substrate-detachment, (e) *Paratetilla arcifera* (voucher specimen [BRKa 005] in underwater), and (f) *P. arcifera* after substrate-detachment.

The study revealed that underwater photos of sponges exhibited notable color differences compared to those taken after removal from their substrate, exposure to air, and preservation in ethanol (Figure 2).

**Figure 3:**
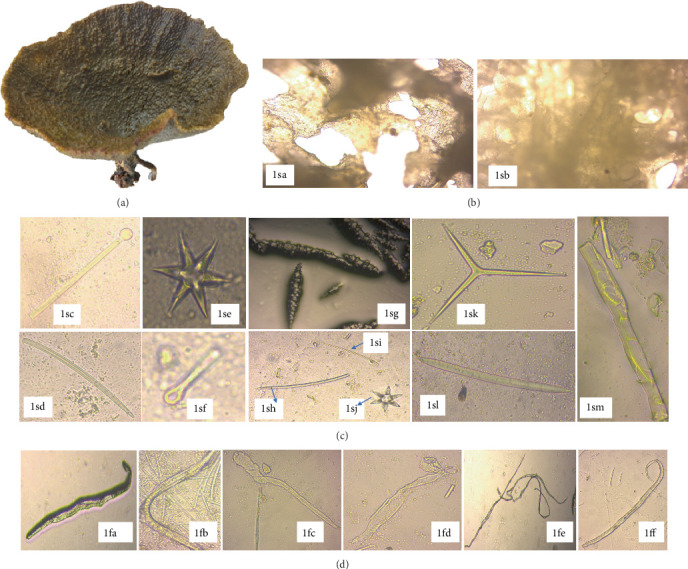
Structural view of *Carteriospongia foliascens*, showing sponge skeleton, spicules, and spongin fibers (40x magnification) (*Source:* Author). Key: (a) CYKu 002—external morphology of *Carteriospongia foliascens*; (b) CYKu 002—skeletal features: 1sa, perpendicular section; 1sb, tangential section; (c) CYKu 002—spicule types: 1sc, tylostrongyles, 1sd and 1sh, styles; 1se, oxyasters; 1sf, knob-end depressed tylostrongyles; 1sg, acanthoxeas; 1si, microxeas; 1sj, micro hexactines; 1sk, triactines; 1sl, oxeas; 1sm, giant basal spicule; and (d) CYKu 002—spongin fibers: 1fa, slightly bent with vertical striations; 1fb, flat twisted; 1fc, collagen-like; 1fd, slightly twisted; 1fe, fully twisted; 1ff, transparent lumen with complete bend (40x magnification).

*C. foliascens*, sampled from Kuruwitu, belongs to the order Dictyoceratida and family Thorectidae. *C. foliascens* exhibited a distinct whorled or curved plate-like structure reaching 15–20 cm in width and height (Figure 3). These “keratose sponges” encompassed shallow environments including mangroves, sandy lagoons, seagrass beds, sandy beaches, and coral reefs. The scattered nodules adorned the surface, each encrusted with sand. The sponges' oscules measuring 1–2 mm were observed on one side of the sponge blade.

The *C. foliascens* displayed significant toughness, with live coloration in their natural habitat ranging from creamy to yellow-whitish. However, upon preservation in ethanol, their color transitioned to a pale white hue. Microscopic examination of the mesophyll revealed collagenous spongin filaments alongside various spicules, including styles (698.0‐1072.3‐1138.7 × 8.7‐18.5‐29.5* μ*m), oxeas (409.0‐496.2‐607.3 × 7.8‐10.2‐17.9* μ*m), tylostrongyles (249.7‐497.1‐806.6 × 3.2‐5‐8.1* μ*m), acanthoxeas (281.7‐467.7‐709 × 5.6‐8.9‐14.9* μ*m), and triactines and giant basal spicules (1125.0‐1518.2‐2031.0 × 10.9‐18.2‐27.4* μ*m). Additional microscleres such as oxyasters, micro hexactines, and microxeas (141.5-167.2-287.7 *μ*m) were also identified in the specimen (Figure 3).

**Figure 4:**
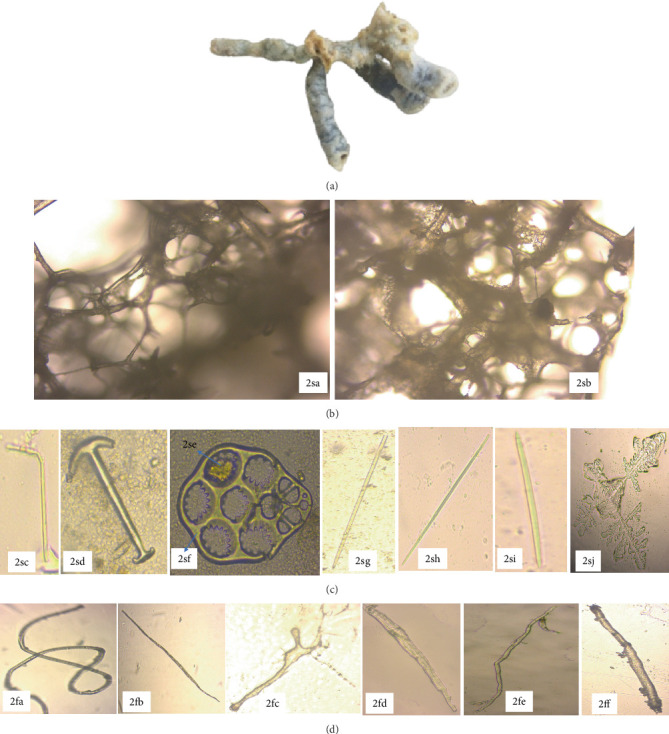
Microstructural composition of *Callyspongia fallax*, highlighting spicules and embedded spongin fibers (40x magnification) (*Source:* Author). Key: (a) GYM 009—external morphology of *Callyspongia fallax*; (b) GYM 009—skeletal framework: 2sa, perpendicular section; 2sb, tangential section; (c) GYM 009—spicule types: 2sc, bend styles; 2sd, anchorates; 2se, miliary granules; 2sf, synapta plates; 2sg and 2sh, styles; 2si, oxeas; 2sj, stauractines megascleres with digit-like projections near tentacles; and (d) GYM 009—spongin fibers: 2fa, coiled fibers with thickened cell walls and transparent lumen; 2fb, simple elongated fibers; 2fc, fibers with irregular cell walls; 2fd, irregular fibers with vertical striations; 2fe, branched spongin fibers; 2ff, fibers with irregular thickened walls (40x magnification).

The marine specimen *C. fallax*, collected from Mtwapa, belongs to the order Haplosclerida and family Callyspongiidae. *C. fallax* exhibited a tubular structure with branching formations (Figure 4). Its primary framework comprised an isodictyal network of spongin fibers intertwined with multiple spicule tracts of diactinal type, creating polygonal patterns. Notable features included conules and a distinct, lace-like network of spongin fibers. The sponge's exterior displayed distinctive ridged sculpting, while its internal choanosomal skeleton showed a looser arrangement with secondary fibers interlaced with numerous synapta plates. Vestigial megascleres were replaced by sand grains, identifiable by their darkened central canals (Figure 4).

The outer skeleton of *C. fallax* featured an array of slender styles measuring (592.0‐870.1‐1232.0 × 17.5‐22.9‐27.2* μ*m). This species typically resided in the shallow, sandy lagoons. The sponge's choanosomal skeleton was observed to be compact, forming an axial structure. The *C. fallax* skeleton comprised a dense multispicular paratangential network of diactinal spicules; hence, it was more compact than the choanosomal skeleton. The latter was distinguished by a network of spongin fibers, ascending and transversely cored multispicular tracts of styles (242.2‐469.0‐798.9 × 4.3‐7.1‐10.3* μ*m). Additionally, erect spicule brushes and stauractines were observed at the surface. The sponge's megascleres were exclusively oxeas (172.2‐188.1‐201.4 × 4.9‐8.4‐11.0* μ*m) interconnected by collagenous spongin, while microscleres were absent. This species typically inhabited the shallow sandy bottoms and mangrove lagoons. The spongin fibers of *C. fallax* encapsulated the core spicules with modified bent styles and were studded with anchorates and synapta plates containing miliary granules. Notably, microscleres were not present in this specimen (Figure 4).

**Figure 5:**
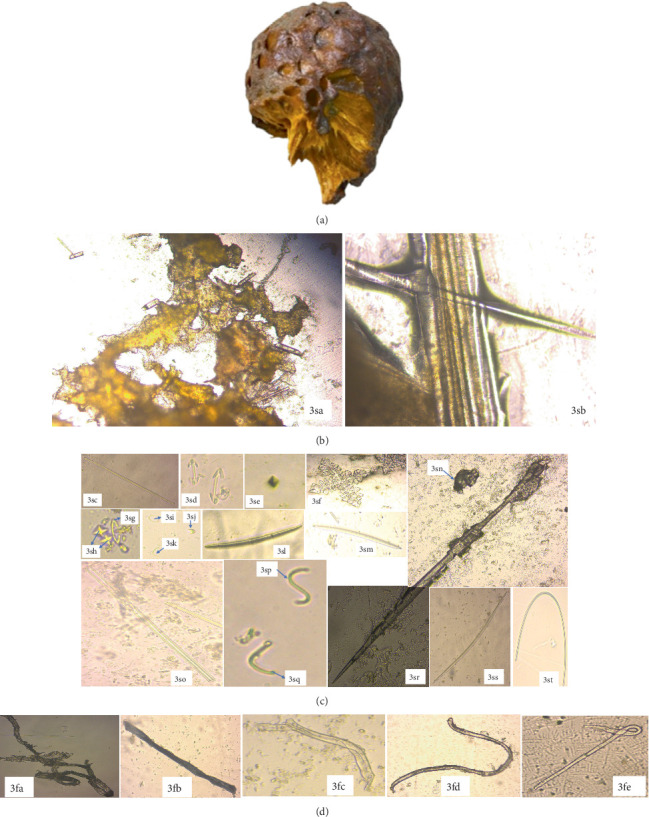
Morphological features of the sponge skeleton in *Paratetilla arcifera*, including spicules and connective spongin fibers (40x magnification) (*Source:* Author). Key: (a) BRKa 005—external morphology of *Paratetilla arcifera*; (b) BRKa 005—skeletal structure: 3sa, perpendicular section; 3sb, tangential section; (c) BRKa 005—spicule types: 3sc, tylotes; 3sd, isochelaes; 3se and 3sh, sterrasters; 3sf, stauractines megascleres with digit-like projections; 3sg, 3si, 3sj, 3sk, 3sq, and 3st, C-shaped sigmas; 3sl, 3sm, 3ss, curved oxeas; 3sn, phytolith artifacts; 3so, tuberculated strongyles with transparent lumen; 3sp, S-shaped sigmas; 3sr, giant basal acanthotylostyle spicule; and (d) BRKa 005—spongin fibers: 3fa, soft coiled fibers; 3fb, black hard elongated fibers; 3fc, twisted and bent fibers; 3fd, fibers with thickened rough cell walls; 3fe, fibers with complete twist at one end (40x magnification).

*P. arcifera*, collected from Kanamai, belongs to the order Tetractinellida and family Tetillidae. The *P. arcifera* sponge had a pear-shaped form with a radial skeletal structure. Its live coloration was yellow–orange, which faded to a pale orange when preserved in ethanol. It featured a stalk and a petal, forming a bellow sponge with a rough surface and nonvisible oscules. The sponge's skeleton was densely packed and composed of long, slender spicules alongside stout sponging fibers. The siliceous spicules consisted of megascleres, including oxeas (1305.0‐1694.2‐2219.0 × 12.3‐17.4‐28.6* μ*m) arranged in a radiated pattern, tylotes, isochelaes, sterrasters, and stauractines. Additionally, other modified spicules were observed, such as the giant basal acanthotylostyles (1667.0‐2158.2‐3089.6 × 9.8‐25.8‐41.7* μ*m) and strongyles (389.2‐571.7‐672.0 × 7.7‐13.7‐28.2* μ*m). The *P. arcifera*'s microscleres comprised two contorted classes of sigma spires: S-shaped (33.2-43.0-66.4 *μ*m) and C-shaped sigmas (31.4-37.1-51.1 *μ*m) with medium spines and (83.7-98.7-117.6 *μ*m) with minute spines. *P. arcifera* was noted to have inhalant pores grouped in special pore areas. These sponges were found inhabiting seagrass beds, shallow sandy lagoons, and sandy beaches (Figure 5).

### 3.2. Taxonomic Affiliation of the Marine Sponges

The sponge specimens were affiliated with the genera *Carteriospongia*, *Callyspongia*, and *Paratetilla* based on percentage sequence similarities and were all classified under the phylum Porifera. A comparative analysis of the newly acquired mitochondrial COI sequences, performed using BLASTn against the GenBank database, revealed sequence similarities of ≥ 99% with established entries in the nucleotide sequence repository (Table 1).

**Figure 6:**
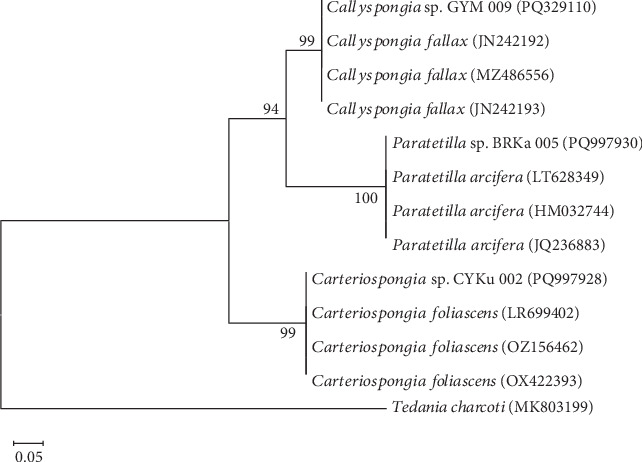
Evolutionary relationships between metazoan COI sequences and some closest known sponge species. *Tedania charcoti* (Accession No. MK803199.1) was used to root the phylogenetic tree. Bootstrap values greater than 50%, derived from 1000 replications, are displayed at branch nodes. The scale bar represents 0.05 substitutions per nucleotide.

The analysis of the three marine sponge specimens (PQ997928, PQ329110, and PQ997930) revealed sequence identity that was ≥ 99% with known species from the nucleotide sequence database. Each specimen clustered distinctly within a separate subcluster, with a bootstrap support value of ≥ 99% (Figure 6 and Table 1). The marine sponge specimens CYKu 002 (PQ997928) from Kuruwitu and BRKa 005 (PQ997930) from Kanamai were closely related to *C. foliascens* and *P. arcifera*, respectively. These specimens exhibited sequence identities of 99% and 100%, respectively (Table 1), and formed subclusters, each supported by a bootstrap value of 99% and 100%, as shown in the phylogenetic tree (Figure 6). The sponge specimen GYM 009 (PQ329110) collected from Mtwapa was affiliated with the known sponge species *C. fallax*, displaying a sequence identity of 100% and forming a subcluster supported by a bootstrap value of 99% (Table 1 and Figure 6).

### 3.3. Antibacterial and Antifungal Activities of the Extracts From Marine Sponges

Extracts from marine sponges were tested for their efficacy against human pathogenic strains: the Gram-positive bacterium *S. aureus* ATCC 27853, the Gram-negative bacteria *E. coli* ATCC 25922 and *P. aeruginosa* ATCC 25923, and the fungus *C. albicans* ATCC 10231 (Tables 2, 3, and 4). The crude organic extracts from *C. fallax* and *C. foliascens* showed significant antimicrobial activity against at least one of the four tested microorganisms, exceeding the efficacy of the positive control (*p* < 0.05) (Tables 2 and 4). The DCM extracts of *C. foliascens* exhibited a higher antifungal activity (31.33 ± 1.2 mm) against *C. albicans* than the positive control (29.33 ± 1.5 mm) (Table 2). The inhibitory activity of EtOAc extracts of *C. fallax* (36.67 ± 0.7 mm) against *S. aureus* was observed to be higher compared to the positive control value of 34.67 ± 0.9 mm, respectively (Table 4). The *P. arcifera* extracts did not exhibit antimicrobial activity when tested against all the test microorganisms (Tables 2, 3, and 4).

### 3.4. Marine Sponge Extract Testing for MICs and the MBCs/MFCs

Ciprofloxacin demonstrated the lowest MIC values: 1.36 mg mL^−1^ against *E. coli* and 1.94 mg mL^−1^ against *P. aeruginosa*. These values were notably lower compared to other drugs tested, including vancomycin (2.00 mg mL^−1^) against *S. aureus* and fluconazole (5.11 mg mL^−1^) against *C. albicans* (Table 5). The MIC values for the organic crude extracts of marine sponges varied from 3.86 ± 0.01 to 5.89 ± 0.01 mg mL^−1^. The EtOAc extract of *C. fallax* showed a MIC of 3.86 ± 0.01 mg mL^−1^ against *S. aureus*, whereas the standard control (vancomycin) had a MIC of 2.00 ± 0.00 mg mL^−1^ (Table 5). For the DCM extract of *C. foliascens*, a MIC of 5.89 ± 0.01 mg mL^−1^ was observed against *C. albicans*, in comparison to the standard drug (fluconazole), which had a MIC of 5.11 ± 0.00 mg mL^−1^ (Table 5).

In all tests, the minimum bactericidal concentrations (MBCs) for ciprofloxacin, vancomycin, and sponge-derived extracts were consistently higher than their MICs (Table 5). The MBC for all reference drugs was determined to be 2.50 mg mL^−1^. The MBC for the EtOAc extracts of *C. fallax* against *S. aureus* was found to be 4.03 mg mL^−1^. The DCM extract of *C. foliascens* demonstrated the most potent fungicidal activity, with a minimum fungicidal concentration (MFC) of 2.50 mg mL^−1^. In contrast, *P. arcifera* and *C. fallax* both demonstrated an MFC of 10 mg mL^−1^.

### 3.5. GC-MS Spectral Analysis of the Crude Extract of the Marine Sponge Extracts

**Figure 7:**
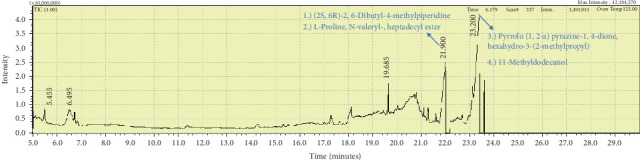
The GC-MS chromatogram analyses of peaks from the dichloromethane extract of *Carteriospongia foliascens* (CYKu 002) showing four potent bioactive compounds.

**Figure 8:**
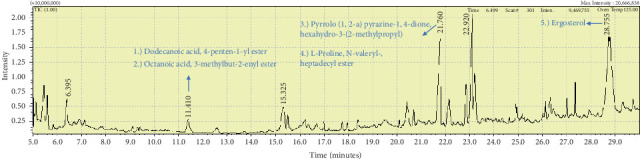
The GC-MS chromatogram analyses of peaks from the ethyl acetate extract of *Callyspongia fallax* (GYM 009) revealed five secondary bioactive compounds.

**Figure 9:**
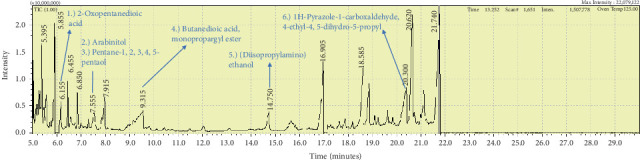
The GC-MS chromatogram analyses of peaks from the methanolic extract of *Paratetilla arcifera* (BRKa 005) demonstrating six nutraceuticals and industrial chemistry compounds.

Two sponge crude extracts (PQ997928 and PQ329110) that exhibited significant antimicrobial activity against at least one of the four tested microorganisms surpassing the efficacy of the positive control, along with one methanolic extract that demonstrated no antimicrobial activity against any of the tested microorganisms, were subjected to GC-MS analysis to identify the compounds present. The GC-MS analysis in our study specifically generated the hit spectrum and chemical structure of the identified compounds (Table 6). The number of different peaks corresponds to the number of compounds (Table 6 and Figures 7, 8, and 9).

The GC-MS chromatogram data revealed the presence of 98 distinct compounds within two bioactive extracts derived from sponges (CYKu2 and GYM 009), as well as in one extract (BRKa 005) that exhibited no bioactivity. The compounds belong to 41 different classes, including acetylated amine alcohols, amines, amides, amide derivatives, amide local anesthetics, amino acid derivatives, amino alcohols, amino esters, amino triazoles, anhydrosugars, aromatic hydrocarbons, barbiturate derivatives, boronic acid derivatives, chiral epoxide alcohols, chloroesters, cyclic acetals, cyclic alkanes, cyclic amides, cyclic amines, cyclic ethers, dicarboxylic acids, diamino compounds, ester derivatives, fatty alcohols, furanone derivatives, glycosides, heterocyclic organic compounds, hydantoin derivatives, morpholide derivatives, nitrosamines, organic acids, phosphinic acid derivatives, piperidine derivatives, polyols, pyrrolidine derivatives, pyrazole derivatives, silyl ether derivatives, steroids, sugar alcohols, thioether alcohols, and thiocyanate esters.

The GC-MS data analysis of the DCM marine sponge extract of *C. foliascens* (CYKu 002) identified a total of 19 chemical components. These include barbiturate derivatives (5.3%), boronic acid derivatives (5.3%), chloroesters (5.3%), cyclic compounds (5.3%), esters (52.6%), furanones (5.3%), heterocyclic compounds (15.8%), and piperidine derivatives (5.3%) (Figure 7).

The EtOAc marine sponge extract of *C. fallax* (GYM 009) exhibited 27 compounds. These compounds include amide derivatives (3.7%), anhydro sugars (7.4%), barbiturate derivatives (3.7%), cyclic ether (3.7%), ester derivatives (44.4%), heterocyclic compounds (18.5%), hydantoin derivatives (3.7%), silyl ether derivatives (3.7%), and steroids (11.1%) (Figure 8).

The GC-MS analysis of the methanolic marine sponge metabolites of *P. arcifera* (BRKa 005) revealed a total of 68 chemical compounds. These compounds were grouped into their chemical classes of acetylated amine alcohols (1.5%), amide local anesthetics (1.5%), amides (1.5%), amines (1.5%), amino acid derivatives (1.5%), amino alcohols (2.9%), amino esters (1.5%), amino triazoles (1.5%), aromatic hydrocarbons (13.2%), chiral epoxide alcohols (4.4%), cyclic acetals (4.4%), cyclic alkanes (1.5%), cyclic amides (1.5%), cyclic amines (5.9%), cyclic ethers (1.5%), dicarboxylic acids (5.9%), diamino compounds (1.5%), esters (17.6%), ether derivatives (7.4%), glycosides (1.5%), morpholide derivatives (1.5%), nitrosamines (1.5%), organic acids (2.9%), phosphinic acid derivatives (1.5%), polyols (2.9%), pyrrolidine derivatives (2.9%), pyrazole derivatives (1.5%), sugar alcohols (4.4%), thioether alcohols (1.5%), and thiocyanate esters (1.5%) (Figure 9).

In our study, the GC-MS chromatogram analysis of the DCM extract from *C. foliascens* (CYKu 002) identified four potent bioactive compounds: 11-methyldodecanol, (2S,6R)-2,6-dibutyl-4-methylpiperidine, L-proline, N-valeryl-, heptadecyl ester, and pyrrolo [1,2-a] pyrazine-1,4-dione, hexahydro-3-(2-methylpropyl) (Figure 7 and Table 6). Similarly, analysis of the EtOAc extract from *C. fallax* (GYM 009) revealed five secondary biochemical compounds including dodecanoic acid, 4-penten-1-yl ester, octanoic acid, 3-methylbut-2-enyl ester, ergosterol, L-proline, N-valeryl-, heptadecyl ester, and pyrrolo[1,2-a] pyrazine-1,4-dione, hexahydro-3-(2-methylpropyl) (Table 6 and Figure 8).

The methanolic extract from *P. arcifera* (BRKa 005) exhibited six nutraceutical and industrial chemistry compounds: 2-oxopentanedioic acid; (diisopropylamino)ethanol; 1H-pyrazole-1-carboxaldehyde, 4-ethyl-4,5-dihydro-5-propyl; butanedioic acid, monopropargyl ester; pentane-1,2,3,4,5-pentaol; and D-arabinitol (Table 6 and Figure 9).

## 4. Discussion

Demosponges are the most diverse class in the phylum Porifera, comprising over 90% of nearly 7000 species worldwide [91]. This study reported three genera (*Carteriospongia*, *Callyspongia*, and *Paratetilla*) within the class Demospongiae. Similar research conducted in the Kalloni Gulf of Lesvos Island, Greece, identified 54 species across 35 genera and 25 families, all within 10 orders of the Demosponge class [92]. In Simeulue Island, Aceh Province, Indonesia, more than 20 species of marine sponges were morphologically characterized and reported, including *C. foliascens* and *P. arcifera* [24].

The morphological results indicated that styles were the most commonly observed spicules, followed by oxeas. Literature indicates that the spicules of Demospongiae exhibit monaxial or tetraxial symmetry, with monaxons such as oxeas and styles being the most prevalent types [93]. Styles and oxeas are fundamental components of the complex skeletons of Demospongiae. These spicules provide structural support and contribute to defense mechanisms against predators and environmental challenges [94]. The identified unique organic spongin fibers in *C. foliascens* form the sponge's fibrous skeleton with a collagen-like substance that makes the sponge a compelling candidate for biomedical applications, including drug delivery and tissue engineering systems [95]. While porcine and bovine sources are the primary producers of collagen for pharmaceutical, cosmeceutical, nutraceutical, and industries, the risk of transmissible diseases such as bovine spongiform encephalopathy is a concern [96]. To date, only a few studies have suggested the potential of marine sponge collagen in biological and biomedical applications [97].

This study utilized COI gene barcoding to confirm the identity of the sponge samples as belonging to three genera, including *Carteriospongia*, *Callyspongia*, and *Paratetilla.* Another study used a multigene approach to assess the systematics of phyllospongiinids in the genera *Carteriospongia*, *Phyllospongia*, and *Strepsichordaia* from tropical Australia and the Red Sea [98]. Elsewhere, a study identified a total of 30 species of Hawaiian sponge fauna using the COI gene, including various species of *Callyspongia* [99]. This confirms that DNA barcoding of the COI gene has emerged as a powerful tool for identifying and classifying marine sponges, particularly in biodiverse and understudied ecosystems [100].

The GC-MS chromatogram data results indicated that two of the sponge samples, which were more effective against at least one of the test microorganisms than the positive control, produced 46 chemical compounds. Marine sponges, especially the class Demospongiae, are fascinating invertebrates due to their bioactive compounds [101]. Marine sponge extracts from *C. foliascens* and *C. fallax* demonstrated notable antimicrobial potency in this study. Bioassay results from a previous study indicated that certain chemicals produced by *C. foliascens* possess antimicrobial activities and have the potential to be used as lead drugs [102]. The DCM extracts of *C. foliascens* in this study showed greater antifungal activity against *C. albicans* compared to the standard drug fluconazole. The potential efficacy of *C. foliascens* against *C. albicans* has also been demonstrated in another research [103]. For the antifungal activity, the compounds identified in this study include ergosterol [77]; 1-(2-ethyl-1,2,4-triazol-3-yl) ethanamine [78]; pyrrolo [1,2-a] pyrazine-1,4-dione, hexahydro-3-(2-methylpropyl) [75]; and dodecanoic acid, 4-penten-1-yl ester [76]. Furthermore, the compound pyrrolo [1,2-a] pyrazine-1,4-dione, hexahydro-3-(2-methylpropyl) found in *C. foliascens* in this study has been reported to exhibit antifungal properties against the soil-borne fungus *Sclerotium bataticola* [79]. Literature also indicates that sponge crude extracts exhibit higher antifungal activity against *C. albicans* compared to standard drugs [104]. These findings confirm that the antifungal activity of *C. foliascens* could have potential therapeutic effects against fungal infections and practical applications in medicine, agriculture, and other fields [80]. Moreover, a study from the northeastern region of Mauritius revealed that the organic extracts from selected sponges exhibited greater antibacterial activity surpassing the standard antibiotic and lower MIC against *S. aureus* and *E. coli* [73].

Among the 98 marine sponge chemical compounds identified in our study, nine have been reported to possess antimicrobial, antibacterial, and antifungal bioactivity properties. Notably, this study has confirmed the presence of previously identified compounds with antimicrobial activity, such as 11-methyldodecanol [77]; (2S,6R)-2,6-dibutyl-4-methylpiperidine [74]; pyrrolo [1,2-a] pyrazine-1,4-dione, hexahydro-3-(2-methylpropyl) [75]; dodecanoic acid, 4-penten-1-yl ester [76]; octanoic acid, 3-methylbut-2-enyl ester [81]; L-proline, N-valeryl-, heptadecyl ester [105]; and ergosterol [77].

A study on the EtOAc extract from the sponges collected from Mauritian waters demonstrated the antibiotic action against *S. aureus* when combined with ampicillin [106]. Our study revealed that organic crude extracts derived from *C. foliascens* and *C. fallax* exhibited antibacterial properties that are effective against one or more bacterial species. Xylitol [104]; 1-(2-ethyl-1,2,4-triazol-3-yl) ethanamine [106]; and pyrrolo[1,2-a] pyrazine-1,4-dione, hexahydro-3-(2-methylpropyl) [75] are known to possess antibacterial properties.

In this study, the methanolic extracts of *P. arcifera* sampled from Kanamai exhibited no bioactivity against the tested microorganisms. However, GC-MS spectral analysis of *P. arcifera* extracts identified several chemical compounds with diverse biological, industrial, and chemical applications beyond bioactivity. These compounds include 2-oxopentanedioic acid, which is utilized in the treatment of metabolic disorders [107], as an athletic performance enhancer [108] and as an intermediate in the Krebs cycle (citric acid cycle) essential for cellular energy production [109]. (Diisopropylamino)ethanol is known for its anticonvulsive and antiaggressive properties [82]. 1H-Pyrazole-1-carboxaldehyde, 4-ethyl-4,5-dihydro-5-propyl plays a role in agricultural chemistry, particularly in the development of pesticides and herbicides [83].

Additionally, butanedioic acid, monomethyl ester, serves as a pharmaceutical excipient in controlled-release formulations [84], a food additive functioning as an acidity regulator and flavor enhancer [39], a cosmetic ingredient used as a pH adjuster and buffering agent, and a key component in the production of succinate-based medications for treating metabolic disorders [89]. Pentane-1,2,3,4,5-pentaol is used as a noncariogenic sweetener and in the synthesis of biodegradable polymers and polyester elastomers for biomedical applications [90], while D-arabinitol serves as a biomarker for detecting *Candida* infections in immunocompromised individuals, indicating systemic candidiasis through elevated serum or urine levels. It is also utilized in research to study enzyme activity, such as D-arabitol dehydrogenase, and as a microbial growth indicator for intestinal overgrowth of *C. albicans* or other yeast/fungal species [87].

Further studies using different solvents, fractionation techniques, and bioassay-guided purification may help isolate and characterize the actual bioactive fractions responsible for antimicrobial activity [88]. Additionally, testing a broader range of pathogens and optimizing extraction conditions may reveal hidden antimicrobial potential [86].

The MIC serial dilutions in this study were utilized to adjust the sponge extract dosage levels, which is essential for evaluating the efficacy of the antimicrobial substances in combating microbial infections [85]. This study's findings reveal that, in comparison to vancomycin and fluconazole, ciprofloxacin exhibited the lowest MIC. A previous study reported that ester derivatives of ciprofloxacin exhibited strong antibacterial activity against penicillin-resistant *E. coli* [110]. This indicates a higher efficacy of ciprofloxacin in inhibiting bacterial growth under the conditions tested in this research.

The higher MBC values across all tested substances indicate that achieving complete bacterial eradication requires greater concentrations than merely inhibiting growth [64]. In our study, while ciprofloxacin and vancomycin exhibited strong bactericidal activity, the EtOAc extract of *C. fallax* also showed promising antimicrobial potential. A previous study investigating EtOAc extracts from *Callyspongia* species identified alkaloid bioactive compounds with MICs of ≥ 128 *μ*g/mL against *Bacillus subtilis*, *E. coli*, *Streptococcus mutans*, and *Salmonella enterica* [111].

This research highlights significant findings regarding the diverse chemical constituents present in the marine sponge extracts, which could have implications for future pharmacological research. The current study results indicate that sponge extracts hold potential as alternatives to conventional antifungal and antibiotic treatments in the field of medicine. Future studies focusing on compound isolation, purification, structural analysis, and mechanisms of action could enhance the efficacy of sponge-derived extracts for medical application in treating human diseases [112].

## 5. Conclusion

This study confirms that marine sponges remain an intriguing area of research for potential antifungal and antibacterial therapies. The extracts from *C. foliascens* and *C. fallax*, which exceeded the efficacy of the positive control against the tested microorganisms in antimicrobial activity, are likely to be therapeutically useful and may contain promising leading molecules for new drugs.

Future research should explore the antiviral potential of sponge-derived bioactive compounds, broadening their application in combating viral pathogens and enhancing their relevance in integrated antimicrobial strategies.

## Figures and Tables

**Table 1:** Affiliation of the metazoan marine sponges with their closest taxonomic relatives.

**Sample ID**	**Accession no.**	**Location**	**Closest taxonomic affiliation**	**Isolation source**	**Country**	**% ID**
CYKu 002	PQ997928	Kuruwitu (3°48⁣′39.6⁣^″^ S 39°49⁣′52.0⁣^″^ E)	*Carteriospongia foliascens* (LR699402)	Reefs [24]	Indonesia	99
GYM 009	PQ329110	Mtwapa (3°57⁣′07.1⁣^″^ S 39°46⁣′05.9⁣^″^ E)	*Callyspongia fallax* (MZ486556.1)	Coral reefs [8]	Zanzibar	100
BRKa 005	PQ997930	Kanamai (3°55⁣′ S 39°46⁣′ E)	*Paratetilla arcifera* (LT628349.1)	Reefs [24]	Indonesia	100

**Table 2:** Antimicrobial activities of dichloromethane organic crude extracts from the selected marine sponges against the tested human pathogenic fungi and bacteria along the Kenyan coastline.

		**Dichloromethane extracts inhibition zone diameters (mm)**			
		**Fungi**	**Gram-negative bacteria**		**Gram-positive bacteria**
**Sample ID**	**Sponge species**	***Candida albicans***	***Escherichia coli***	***Pseudomonas aeruginosa***	***Staphylococcus aureus***
CYKu 002	*Carteriospongia foliascens*	31.33 ± 1.2^abc^	26.00 ± 1.2^a^	23.33 ± 2.6^abcde^	28.67 ± 1.8^a^
GYM 009	*Callyspongia fallax*	8.33 ± 0.3^bc^	10.33 ± 0.9^fg^	8.67 ± 0.3^fg^	10.00 ± 0.6^cde^
BRKa 005	*Paratetilla arcifera*	0.00 ± 0.0^c^	0.00 ± 0.0^g^	0.00 ± 0.0^g^	0.00 ± 0.0^e^
Positive control		29.33 ± 1.5^ab^	31.67 ± 0.7^ab^	31.22 ± 0.6^ab^	31.11 ± 0.2^ab^
Negative control		0.00 ± 0.0^c^	0.00 ± 0.0^g^	0.00 ± 0.0^g^	0.00 ± 0.0^e^

**Table 3:** Antimicrobial activities of methanolic organic crude extracts from the selected marine sponges against the tested human pathogenic fungi and bacteria from the Kenyan waters.

		**Methanolic extracts inhibition zone diameters (mm)**			
		**Fungi**	**Gram-negative bacteria**		**Gram-positive bacteria**
**Sample ID**	**Sponge species**	***Candida albicans***	***Escherichia coli***	***Pseudomonas aeruginosa***	***Staphylococcus aureus***
CYKu 002	*Carteriospongia foliascens*	0.00 ± 0.0^d^	16.00 ± 4.0^cd^	12.67 ± 2.6^abc^	18.33 ± 2.4^abcd^
GYM 009	*Callyspongia fallax*	8.33 ± 0.3^bcd^	9.67 ± 0.7^cd^	9.00 ± 0.6^abc^	9.67 ± 0.7^bcd^
BRKa 005	*Paratetilla arcifera*	0.00 ± 0.0^d^	0.00 ± 0.0^d^	0.00 ± 0.0^c^	0.00 ± 0.0^d^
Positive control		26.67 ± 0.3^ab^	27.67 ± 0.9^a^	31.22 ± 0.6^a^	31.11 ± 0.2^abc^
Negative control		0.00 ± 0.0^d^	0.00 ± 0.0^d^	0.00 ± 0.0^c^	0.00 ± 0.0^d^

**Table 4:** Antimicrobial activities of ethyl acetate organic crude extracts from the selected marine sponges against the tested human pathogenic fungi and bacteria along the coastline of Kenya.

		**Ethyl acetate extracts inhibition zone diameters**			
		**(mm)**			
		**Fungi**	**Gram-negative bacteria**		**Gram-positive bacteria**
**Sample ID**	**Sponge species**	***Candida albicans***	***Escherichia coli***	***Pseudomonas aeruginosa***	***Staphylococcus aureus***
CYKu2	*Carteriospongia foliascens*	0.00 ± 0.0^c^	0.00 ± 0.0^d^	14.00 ± 2.1^e^	9.33 ± 0.9^d^
GYM 009	*Callyspongia fallax*	13.67 ± 2.4^bc^	17.33 ± 6.1^b^	15.67 ± 0.3^abcde^	36.67 ± 0.7^abcd^
BRKa 005	*Paratetilla arcifera*	0.00 ± 0.0^c^	0.00 ± 0.0^d^	0.00 ± 0.0^e^	0.00 ± 0.0^d^
Positive control		26.67 ± 0.3^ab^	27.67 ± 0.9^a^	31.22 ± 0.6^abcd^	34.67 ± 0.9^ab^
Negative control		0.00 ± 0.0^c^	0.00 ± 0.0^d^	0.00 ± 0.0^e^	0.00 ± 0.0^d^

**Table 5:** Minimum inhibitory concentrations (MICs) of selected marine sponge extracts against human pathogenic fungi and bacteria from the Kenyan waters.

			**MIC (mg mL** ^**−1**^ ** )**			
**Sample ID**	**Marine sponge**	**Extract**	***Candida albicans***	***Escherichia coli***	***Pseudomonas aeruginosa***	***Staphylococcus aureus***
CYKu2	*Carteriospongia foliascens*	Dichloromethane	5.89 ± 0.01^a^	—	—	—
GYM 009	*Callyspongia fallax*	Ethyl acetate	—	—	—	3.86 ± 0.01^a^
Positive control			5.11 ± 0.00^a^	1.36 ± 0.00^a^	1.94 ± 0.00^a^	2.00 ± 0.00^a^
Negative control			0.00 ± 0.00	0.00 ± 0.00	0.00 ± 0.00	0.00 ± 0.00

**Table 6:** Nature and antimicrobial properties of some of the marine natural products from *Carteriospongia foliascens*, *Callyspongia fallax*, and *Paratetilla arcifera* extracts from the Kenyan coastline identified by the GC-MS chromatogram data in this study.

**Sponge extract source**	**Type of compounds**	**Retention time (minutes)**	**Compound**	**Mass spectrum and chemical structure of the bioactive compounds**	**Molecular formula**	**Molecular weight (g/mol)**	**Quality of similarity (%)**	**Bioactivity**
*Carteriospongia foliascens* and *Callyspongia fallax*	L-Proline derivatives	21.900	L-Proline, N-valeryl-, heptadecyl ester	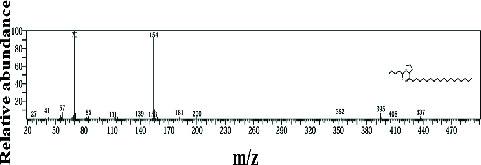	C_27_H_51_NO_3_	437	79	Antiviral activity in plants [73] and antitumor agents [74].
*Carteriospongia foliascens* and *Callyspongia fallax*	Pyrrolidine derivatives	21.900	Pyrrolo [1,2-a] pyrazine-1,4-dione, hexahydro-3-(2-methylpropyl)	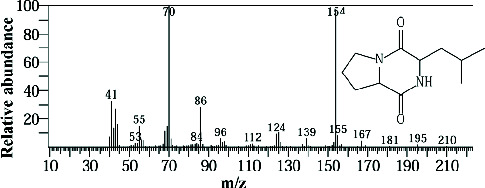	C_11_H_18_N_2_O_2_	210	80	Antifungal [75] and antioxidant and antibacterial activity [76].
*Carteriospongia foliascens*	Fatty alcohols	23.200	11-Methyldodecanol	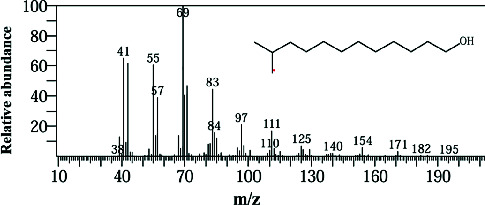	C_13_H_28_O	200	83	Antibacterial activity against *Mycoplasma pneumoniae* and *Helicobacter pylori* [77].
*Carteriospongia foliascens*	Cyclic amines	23.200	(2S,6R)-2,6-Dibutyl-4-methylpiperidine	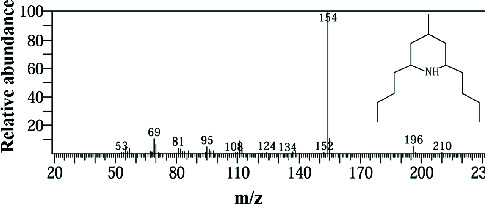	C_14_H_29_N	211	79	Antimicrobial activity; inhibits topoisomerase II (DNA gyrase) and topoisomerase IV [78].
*Callyspongia fallax*	Fatty acid esters	11.410	Dodecanoic acid, 4-penten-1-yl ester	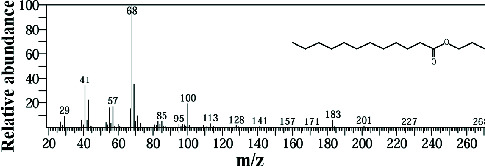	C_17_H_32_O_2_	268	80	Antimicrobial, antifungal, anti-inflammatory, and antiviral properties [79].
*Callyspongia fallax*	Fatty acid esters	11.410	Octanoic acid, 3-methylbut-2-enyl ester	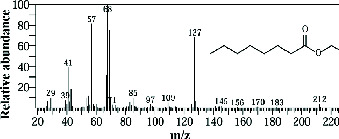	C_13_H_24_O_2_	212	79	Antimicrobial and anti-inflammatory properties [80].
*Callyspongia fallax*	Steroids	28.755	Ergosterol	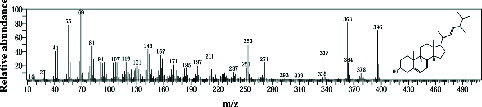	C_28_H_44_O	396	88	Antifungal, vitamin D2 production, nutritional value, and pharmacological activities [81].
*P. arcifera*	Alpha-ketoglutaric acids	6.155	2-Oxopentanedioic acid	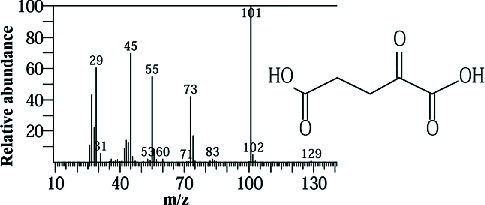	C_5_H_6_O_5_	146	83	Metabolic disorders treatment [82], athletic performance enhancer [83], and Krebs cycle (citric acid cycle) intermediate for energy production in cells [84].
*P. arcifera*	Sugar alcohols	7.555	D-Arabinitol	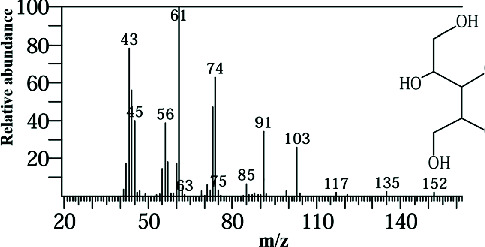	C_5_H_12_O_5_	152	76	A biomarker used to detect *Candida* infections in immunocompromised individuals, indicates systemic candidiasis through elevated serum or urine levels, aids in research by studying enzyme activity like D-arabitol dehydrogenase, and serves as a microbial growth indicator for intestinal overgrowth of microbes such as *Candida albicans* or other yeast/fungus species [85].
*P. arcifera*	Polyols	7.555	Pentane-1,2,3,4,5-pentaol	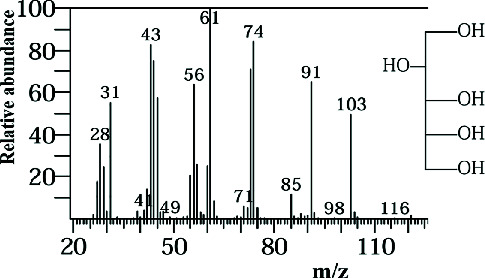	C_5_H_12_O_5_	152	78	A noncariogenic sweetener in the synthesis of biodegradable polymers and polyester elastomers for biomedical applications [86].
*P. arcifera*	Ergot alkaloids	9.315	Butanedioic acid, monopropargyl ester	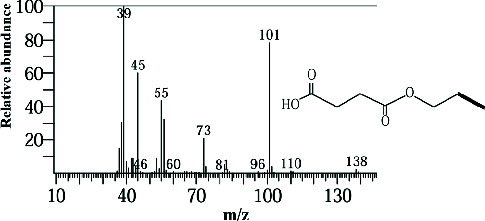	C_7_H_8_O_4_	156	85	Pharmaceutical excipient (in controlled-release formulations) [87]; food additive (acidity regulator and flavor enhancer) [39]; and cosmetic ingredient (pH adjuster and buffering agent), and in the production of succinate-based medications for treating metabolic disorders [88].
*P. arcifera*	Amino alcohols (ethanolamines)	14.750	(Diisopropylamino)ethanol	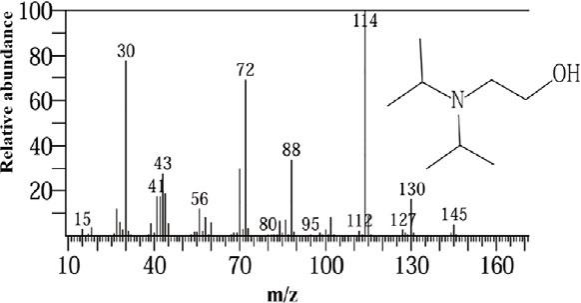	C_8_H_19_NO	145	83	Anticonvulsive and antiaggressive properties [89].
*P. arcifera*	Aromatic heterocyclic organic compound (pyrazole derivative)	20.300	1H-Pyrazole-1-carboxaldehyde, 4-ethyl-4,5-dihydro-5-propyl	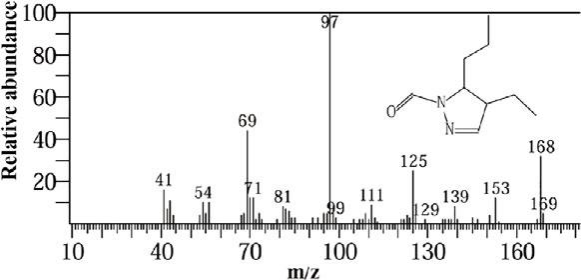	C_9_H_16_N_2_O	168	78	Agricultural chemistry in the development of pesticides and herbicides [90].

## Data Availability

The data presented in this study are available upon request from the corresponding author.

## Supporting Information

Supporting Information Additional supporting information can be found online in the Supporting Information section. Table S1: Summary of marine sponge species observed in high abundance (≥ 4 sites) across Kenyan coastal study areas. Table S2: Summary of marine sponge species recorded at moderate abundance (two to three sites) along the Kenyan coastline. Table S3: Summary of marine sponge species observed at rare abundance (single site) along the Kenyan coastline.
