# Supplementary material for: Identification and Antimicrobial Potential of Marine Sponges (Carteriospongia foliascens, Callyspongia fallax, and Paratetilla arcifera) from Kenyan Marine Waters
Source: Int J Microbiol. 2025 Sep 3;2025:4208163. doi: 10.1155/ijm/4208163 (PMC12433740; doi:10.1155/ijm/4208163)
Supplement: Supporting Information — Additional supporting information can be found online in the Supporting Information section. Table S1: Summary of marine sponge species observed in high abundance (≥ 4 sites) across Kenyan coastal study areas. Table S2: Summary of marine sponge species recorded at moderate abundance (two to three sites) along the Kenyan coastline. Table S3: Summary of marine sponge species observed at rare abundance (single site) along the Kenyan coastline. [file 4208163.f1.docx]

# **Manuscript 4208163**

**Identification and** **Antimicrobial Potential of Marine Sponges (*Carteriospongia foliascens*, *Callyspongia fallax*, and *Paratetilla arcifera*)** **from Kenyan marine waters**

**Supporting information**

**S1 Table:** Summary of marine sponge species observed in high abundance (≥4 sites) across Kenyan coastal study areas

| **Sponge Species (Taxon)** | **Morphotype / Color** | **Sites** | **Habitat Notes** |
| --- | --- | --- | --- |
| *Carteriospongia foliascens* | Fan-shaped / Green | Kuruwitu, Kanamai, Sii Island, Mtwapa Creek, Mundini and Ras Kiromo | Found on mangroves, sandy lagoons, seagrass beds, sandy beaches, and coral reefs |
| *Callyspongia diffusa* | Tube-like / Brown |  | Common in seagrass beds and coral reef slopes; tolerates turbid shallow waters |
| *Callyspongia plicifera* | Tube-like / Orange |  | Often found on coral rubble and protected reef flats |
| *Callyspongia siphonella* | Tube-like / Cream |  | Typically occurs in shallow reef lagoons and sheltered bays |
| *Haliclona tubifera* | Encrusting / Green |  | Commonly associated with mangrove roots and soft-bottom habitats |
| *Haliclona oculata* | Encrusting / Brown |  | Attached to rocks and stones on sandy or rocky substrate |

**S2 Table:** Summary of marine sponge species recorded at moderate abundance (2–3 sites) along the Kenyan coastline

| **Sponge Species (Taxon)** | **Morphotype / Color** | **Sites** | **Habitat Notes** |
| --- | --- | --- | --- |
| *Biemna fistulosa* | Encrusting / Brown | Ras Kiromo and Mtwapa Creek | Present along sandy shores and in mangrove lagoons |
| *Tedania charcoti* | Pear-shaped / Brown-Orange | Sii Island, Mtwapa Creek and Ras Kiromo | Shallow tropical waters, near mangrove roots and sandy lagoons |
| *Callyspongia ramosa* | Tube-like / Orange-Grey | Kanamai and Mtwapa Creek | Grows on reef crests and within shallow lagoons |
| *Callyspongia pseudotoxa* | Tube-like / Cream-Green | Kuruwitu, Kanamai and Mtwapa Creek | Found on sandy substrates and patch reef systems |
| *Haliclona implexiformis* | Encrusting / Purple |  | Typically associated with mangrove channels or soft-sediment substrates |
| *Haliclona stilensis* | Encrusting / Brown | Kuruwitu, Mtwapa Creek and Sii Island | Usually encrusts seagrass blades, rubble, or coral fragments |
| *Haliclona cinerea* | Encrusting / Purple | Kuruwitu, Kanamai, Ras Kiromo | Inhabits intertidal rock pools and coral reef flats |
| *Callyspongia fallax* | Tube-like / Grey | Mtwapa Creek, Mundini | Found on shallow sandy bottoms and in mangrove lagoons |
| *Haliclona laubenfelsi* | Encrusting / Cream-Green | Sii Island, Mundini, Ras Kiromo | Found in protected lagoons and among coral assemblages |
| *Clathria rugosa* | Fan-shaped / Brown |  | Grows on coral reef slopes and rubble zones |
| *Clathria reinwardti* | Fan-shaped / Red |  | Occurs in reef crevices and shaded lagoon habitats |
| *Clathria prolifera* | Fan-shaped / Orange |  | Found on rocky reef faces and in tide pools |
| *Clathria parthena* | Fan-shaped / Brown |  | Typically inhabits shallow coral reefs and rubble beds |

**S3 Table:** Summary of marine sponge species observed at rare abundance (single site) along the Kenyan coastline

| **Sponge Species (Taxon)** | **Morphotype / Color** | **Sites** | **Habitat Notes** |
| --- | --- | --- | --- |
| *Stylissa carteri* | Fan-shaped / Orange | Sii Island | Found in mangrove lagoons, coral reef margins, and sandy beach zones |
| *Agelas cerebrum* | Tube-like / Brown | Mtwapa Creek | Typically found in cryptic reef environments, attached to hard substrata |
| *Haliclona fascigera* | Tube-like / Blue | Ras Kiromo | Shallow sandy bottoms of lagoons and coral reef vicinities |
| *Paratetilla arcifera* | Pear-shaped / Brown | Kanamai | Inhabits seagrass beds, shallow sandy lagoons, and sandy beaches |
